# A serious game for improving the decision making skills and knowledge levels of Turkish football referees according to the laws of the game

**DOI:** 10.1186/s40064-016-2227-0

**Published:** 2016-05-14

**Authors:** Ulas Gulec, Murat Yilmaz

**Affiliations:** Department of Computer Engineering, Çankaya University, Ankara, Turkey

**Keywords:** Digital game-based learning, Interactive learning environments, Serious games

## Abstract

Digital game-based learning environments provide emerging opportunities to overcome learning barriers by combining newly developed technologies and traditional game design. This study proposes a quantitative research approach supported by expert validation interviews to designing a game-based learning framework. The goal is to improve the learning experience and decision-making skills of soccer referees in Turkey. A serious game was developed and tested on a group of referees (N = 54). The assessment results of these referees were compared with two sample t-test and the Wilcoxon signed-ranked test for both the experimental group and the control group. The findings of the current study confirmed that a game-based learning environment has greater merit over the paper-based alternatives.

## Introduction

Football is the world’s most popular sport with approximately 33.4 million individuals involved  (Giulianotti and Robertson 2004). It has a huge business segment which is driven by a significant economic force that had expanded surprisingly in the early 1990s (Dobson and Goddard 2011). Ultimately, the game produces a significant economic impact (i.e. jobs and income) in many countries. Most importantly, referees are an essential part of the game. They are the decision-makers of football matches. It has been observed that the average number of decisions in 31 matches, which were played during EURO 2000, was 137 per match (Helsen and Bultynck 2004); however, nearly 25 percent of these decisions were incorrect. Another study showed that 17 percent of the decisions were incorrect at the 1986 World Cup in Mexico (Van Meerbeek et al. 1987). These studies show that not all referees do their job well, and ultimately their decision-making skills ought to be improved.

Although referees need to be coached and advised as they progress through their careers, there are only a limited number of resources dedicated to training novice football referees. In fact, the *International Federation of Association Football’s (FIFA) Laws of the Game* (LOG) book consists of the rules of the game of football (FIFA 2014). Traditionally, football referees are expected to study this book so as to improve their levels of knowledge. However, using the LOG book has some disadvantages.

Although the LOG book consists of the rules of football, it does not include any information that is useful for improving the decision-making skills of football referees. In particular, it includes textual information; however, many of the previous studies have suggested that using multimedia tools might be beneficial for improving decision-making skills (Savoldelli et al. 2006; Trevena et al. 2006; Volandes et al. 2009). For example, Plessner et al. (2009) conducted a study to increase the number of accurate decisions made by referees in the course of a match. The results show that multimedia technologies, such as videos, do indeed enhance the decision-making skills of referees.

One disadvantage is that reading books is not an interesting activity for males since most of the referees in the Turkish Professional Leagues are males. From a sociocultural perspective, females are more interested in studying from a book than males (Ellis et al. 2008). In support, a study analyzed the reading habit of students at the Pamukkale University School of Sports Science and Technology. The results show that female students appear to be more likely to study from books (Arslan et al. 2009).

According to these findings, the football referees may not be sufficiently able to improve themselves with regard to the rules of football if they were to choose the LOG book as a study tool. Consequently, their decision-making skills are not sufficiently advanced, which may lead to more debatable decisions in football matches. It may be claimed that a small number of referee errors might not be considered very critical in a football match; however, there is the possibility of several consequences of such an outcome. For example, team managers, players and supporters would aggressively protest a referee if there is a controversial decision during a match. Occasionally, such aggressive behavior may even directly target referees [e.g. being rushed and killed (Adinkrah 2012; Hay 1999)]. In order to avoid these undesirable situations, authors claim that interactive game-based training ought to be implemented in order to improve the quality of referees’ decisions.

In light of these remarks, the aim of this study is to develop a serious game, thus assisting football referees to assess their level of knowledge regarding the rules of the game. Consequently, this study is used to advance referee decision-making skills. Such a system needs to be based on text-based questions which assess the participants’ knowledge concerning the rules of football. However, it also requires multimedia questions. Such questions may show the critical positions in a match to test the skills of referees. Thus, the system not only offers practical information that improves the level of knowledge of individuals regarding the rules of football, it also suggests a way for improving the decision-making abilities of football referees.

The overall structure of the study is formed as follows: section “Background and related works” begins with a review of the literature. Section “Methods” explains the methodology of the research, the mechanism of the game, game dynamics, game rules and system description. In section “Experiments”, the two conducted experiments are explained in detail followed by a presentation, analysis and discussion of the results of these experiments. The final section explains the conclusion and the future plans related to this study.

## Background and related works

The main idea behind digital game-based learning is to educate participants by using a video game (Prensky 2007). The goal is to improve the learning experience by utilizing a set of game elements to foster interactivity (Kiili 2005; Pivec 2004). In the literature, there are a number of studies that benefits from the useful properties of games to train individuals in various areas (Farrow and Abernethy 2002; Kuo 2007; Mascarenhas et al. 2005; Papastergiou 2009; Schweizer et al. 2011; Xian 2011; Yang 2011).

Sport is one of the areas in which the advantages of computer technology are found to be useful when instructing participants. An example is Yang (2011), who developed a visual referee learning system for volleyball referees where referees can access and answer the questions prepared by volleyball experts. As a result, this system brings advantages to referees such as improvement in their knowledge levels and ultimately enhance their decision-making skills. Mascarenhas et al. (2005) is another example which improves developing rugby referees’ performance by utilizing video recordings from real rugby games. The goal is to promote a shared mental model training approach. In particular, members of an experimental group have opportunities to receive experts’ explanations on positions, which, as results indicate that developing referees have major advantages for enhancing their decision-making skills.

Farrow and Abernethy (2002) developed an application to help the training of junior tennis players. The aim is to acquire a prediction ability concerning how a player should shoot the ball with intended properties, including properties such as speed, direction and shooting style. Players are divided equally into a number of groups. A selected group watches videos and receives feedback from each video. The final results illustrate that those players who watched the video improved their shooting ability more quickly than those players who were not involved in the interactive training process.

There are a few studies to improve the decision-making skills of football referees. Catteeuw et al. (2010) signifies that more than 10 percent of offside decisions were incorrect at the 2002 and 2006 World Cups. In order to reduce this ratio, a computer-based tool as video has been developed that demonstrates offside positions to the 57 assistant referees. The results suggest that all assistant referees are able to improve their decision-making abilities. Xian (2011) considers soccer referees to be a comprehensive evaluation area which includes the division line, competition skills and knowledge level of the rules of the game for referees. The aim was to develop a web-based multimedia teaching system that educates referees in the rules of football competition. According to the results of cognitive test and post-test, this approach can be used to improve the teaching process. Schweizer et al. (2011) designed a video-based training tool that improves the accuracy rate of soccer referees’ decisions. Referees are incorporated into a system, view videos, make decisions and collect feedback and results. According to their results, the decision-making skills of soccer referees advance in a positive manner.

In summary, there are several studies in the literature that examine game technologies to educate learners in a variety of domains. Although there is no study that benefits from game design techniques to improve the knowledge levels and decision-making skills of the football referees, studies in various domains suggest that they may provide great benefits for the training of football referees.

## Methods

Many researchers have utilized a tailored research methodology to provide a comprehensive understanding of a complex research problem especially when it is important to observe relevant results that may have been obtained from both qualitative and quantitative viewpoints. Here, a quantitative research approach supported by expert validation interviews was employed since it was necessary to have flexibility in order to gather the opinions of referees so as to combine a group of participants’ expectations of the system. Their opinions added confidence to our approach and some suggestions led to improvement of the system. The primary users for such a system were novice referees; therefore, a quantitative survey (i.e. administering pre-test and post-test and analyzing their results) was essential where it was important to assess their requirements and monitor their progress. In addition, it was important to include a qualitative aspect in this study as the viewpoints of referees were crucial to address the design challenges during the development of such an interactive software-intensive system.

For the qualitative part of the study, expert reviews were conducted on five experienced referees who had been working in the education department of the Turkish Football Federation for at least 10 years when a database of 700 questions for the game platform was prepared. The protocol used for the expert reviews were based on the following questions “Do you think this question is suitable for assessing a novice referee?”, “Can you please rate this question between 1 and 3 in terms of its difficulty level?”, “What would be learning outcome for this question?”, “Can the referees reach this question in the other platforms?” and “Is this question similar to the questions that are used in your training programs?”.

For the final qualitative phase of the study, validation interviews were conducted with the participants so as to explore the benefits and potential issues encountered during the interactive assessments by asking these questions:

- **Q1:** Do you think you can improve your knowledge level and decision-making skills by playing the game?
- **Q2:** Which question category/categories are more useful for you?
- **Q3:** Do you prefer to play the game or study from the FIFA book?
- **Q4:** What kind of features do you think are missing in the training framework?
- **Q5:** Do you like to have a competition between the other players? If your answer is yes, is it useful or not?

The answers were evaluated and categorized as positive, negative or neutral by the five retired first class football referees (who had managed matches in the Super League for at least 7 years).

For the quantitative part of the study, an interactive assessment (i.e. pre-test and post-test) was conducted in the form of a game-like structure to a group of football referees, namely those who have joined the referee training program. The aim was to explore the benefits of the proposed platform in improving the knowledge level and decision-making skills of these football referees by measuring the differences between the referees’ pre-test and post-test scores. The numerical results of both tests were analyzed by using statistical techniques to understand whether or not a significant difference between the groups exists.

### Design and implementation

A serious game is a special type of game which is used to educate (or sometimes inform) participants about a selected topic (Abt 1987). Here, a trivia-like game (i.e. interactive assessment) with a race to an end game dynamic (i.e. a pattern of play) is proposed. The idea is for the player to reach the end of the board as quickly as possible. In other words, the participants are to complete the racecourse within a time constraint.

The game has three different types of questions related to the laws of the game of football, which are: (1) True/False Questions, (2) Multiple Choice Questions and (3) Video Questions. The questions in the *True/False Questions* and *Multiple Choice Questions* categories are selected from the actual questions that are administered every year to measure a referee’s level of knowledge of the rules of football while the questions in the *Video Questions* category consist of videos of critical positions that occur in real matches that were played during past football seasons.

**Fig. 1:**
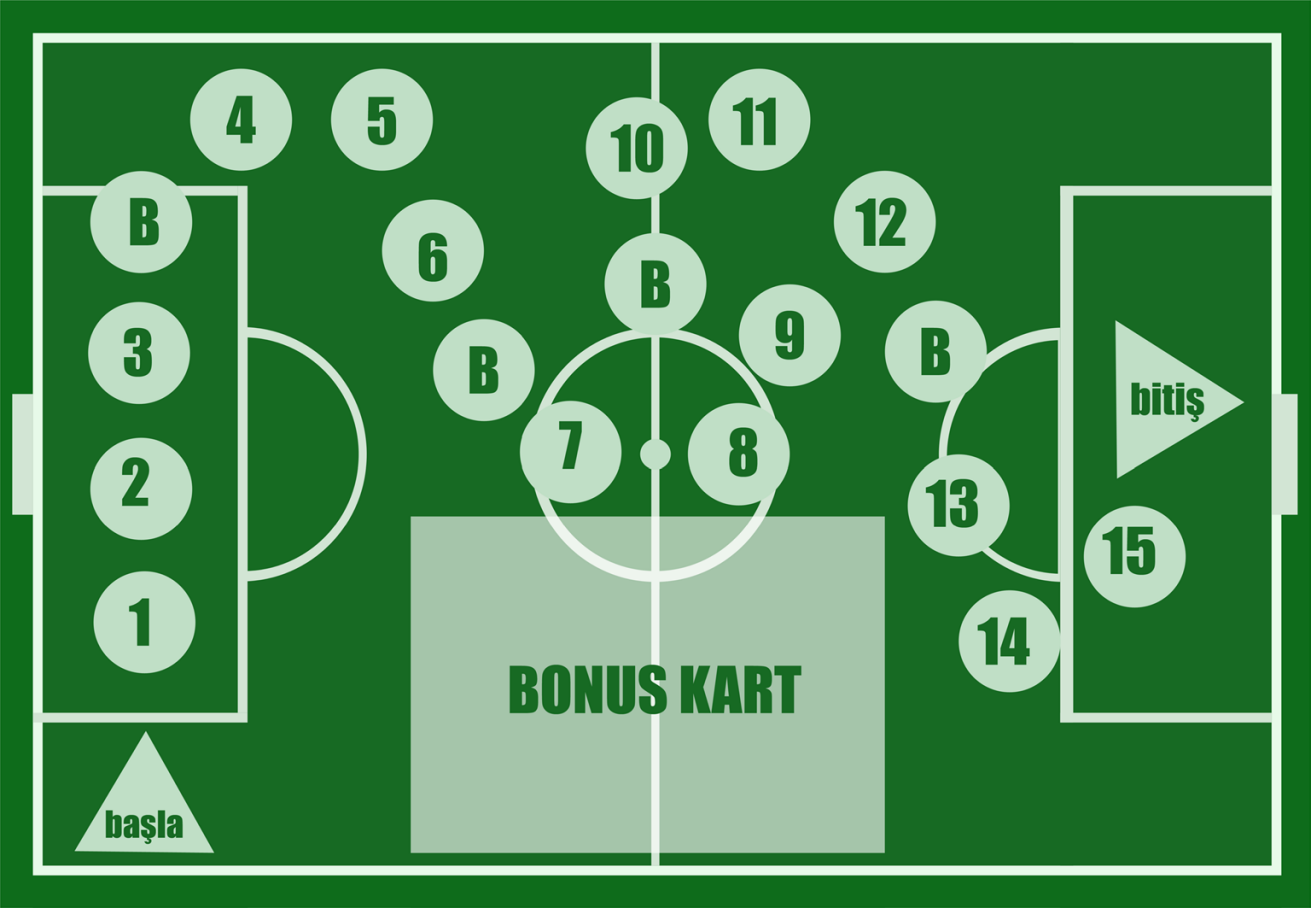
Board of the game

In addition to the question categories, a virtual game board was designed. The game board consists of a set of steps as shown in Fig. 1.

Using a traditional perspective, the participants use a virtual dice with three faces which are corresponding with three numbers. Each number on the die represents each question category as follows: (1) represents True/False Questions, (2) represents Multiple Choice Questions, and (3) represents Video Questions.

**Fig. 2:**
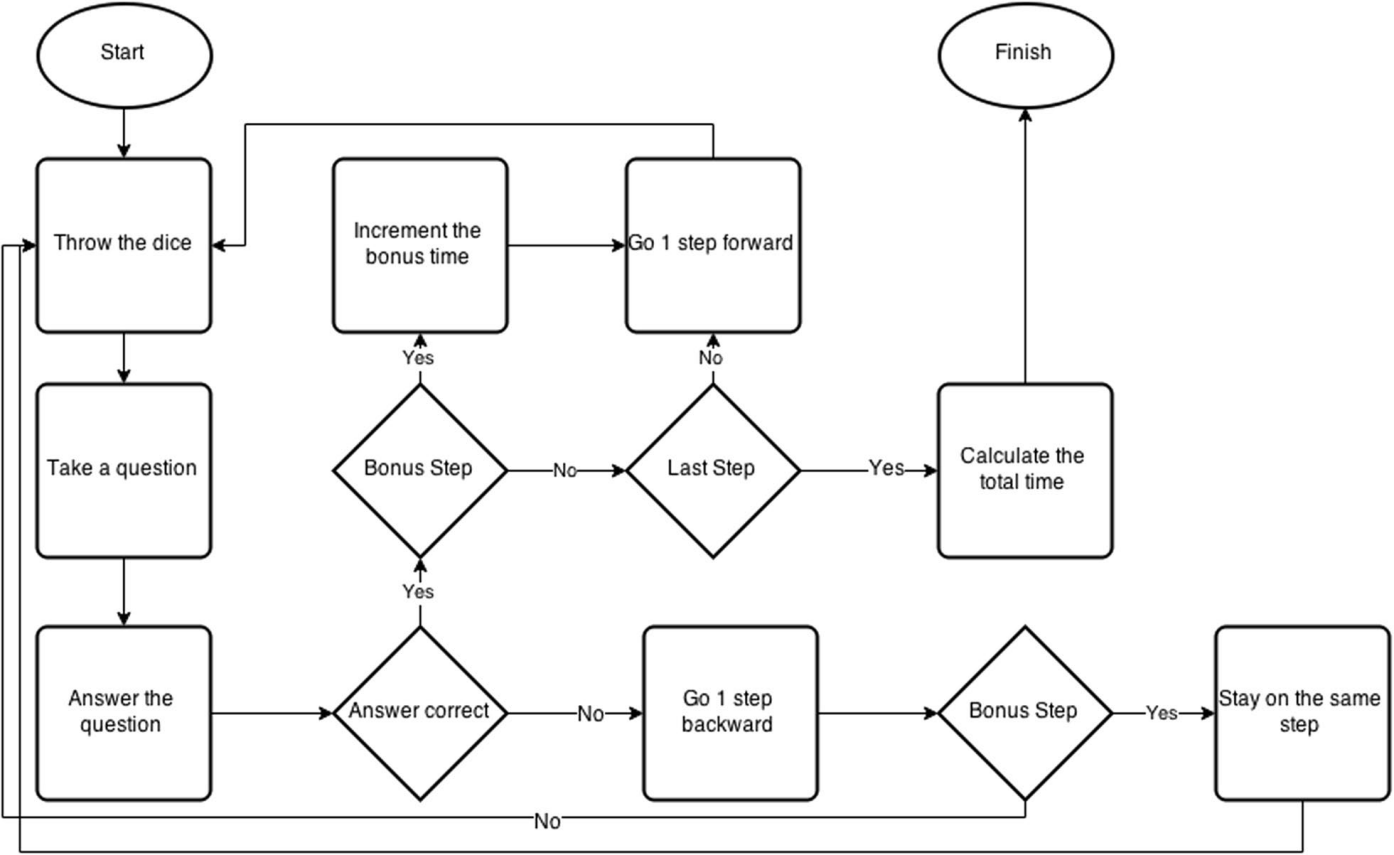
The systematic flow of the game

The mechanics of the game can be identified as follows:

- First, the player throws the die, and produces a one, two or three. Based on the number thrown, he takes a question from one of the corresponding categories above. For example, if the die shows 3, the player takes a question from the video questions category.
- If the player answers correctly to the question, he moves one step forward. Otherwise, he moves one step backward (unless the player is at the starting point or at a bonus step). Before going one step backward, the correct answer to the questions and a detailed explanation of the correct answer is shown to the player.
- As a simple reward structure, there is a group of checkpoints in the game known as bonus steps. The bonus steps are designed to keep the player in the game. This also prevents players from answering incorrectly to a group of questions and returning to the starting point. In addition, this mechanism incrementally provides the player with small rewards thereby achieving motivation.
- The bonus steps also offer additional time for correct answers to a question being asked in this step. The first bonus step gives 5 s of bonus time, the second bonus step offers 10 s, the third bonus step offers 15 s, and finally 20 s are added as bonus time for the fourth bonus step.
- The timer of the game starts with the first question and it stops when a player answers the final question.
- Finally, each player’s racecourse completion time is compared and placed in a table which sorts the players according to their completion time. The player at the top of the leader-board is the winner. The flow of the game is illustrated in Fig. 2.

## Experiments

To evaluate the effect of our game correctly, we conducted experiments in which we allowed participants play the game. Following this, we analyzed the results of these experiments. Creating a sample is necessary in order to obtain valid results. The members of the sample group are selected to have a similar level of knowledge at the beginning of the training program. In order to ensure this similarity, 54 football referees, all of whom had completed the referee course at the same time, were selected. These referees were randomly separated into a control group (N = 27) and an experimental group (N = 27). The referees in the control group are not allowed to log in to our game system. They were only able to learn the rules of the game from the LOG book. The referees in the experimental group were allowed to log in to our game system. However, they were not permitted to use LOG book.

In order to evaluate the level of knowledge of the referees regarding the game rules, a pre-test was administered to a selected group of referees for the training program at the beginning of the training period. This test consisted of 50 questions, which were separated into two sections thus:

- Multiple Choice Questions
- Video Questions

From a group of questions, 35 multiple choice questions and 15 video questions were selected.

To build a proper assessment tool, an expert from the Turkish Football Federation was interviewed. The number and content of questions in the test was determined by him. This expert indicated that there are 50 questions in the real exam administered by the Turkish Football Federation to measure a referee’s level of knowledge of the rules of the game. Hence, the number of questions determined in both tests was 50.

**Fig. 3:**
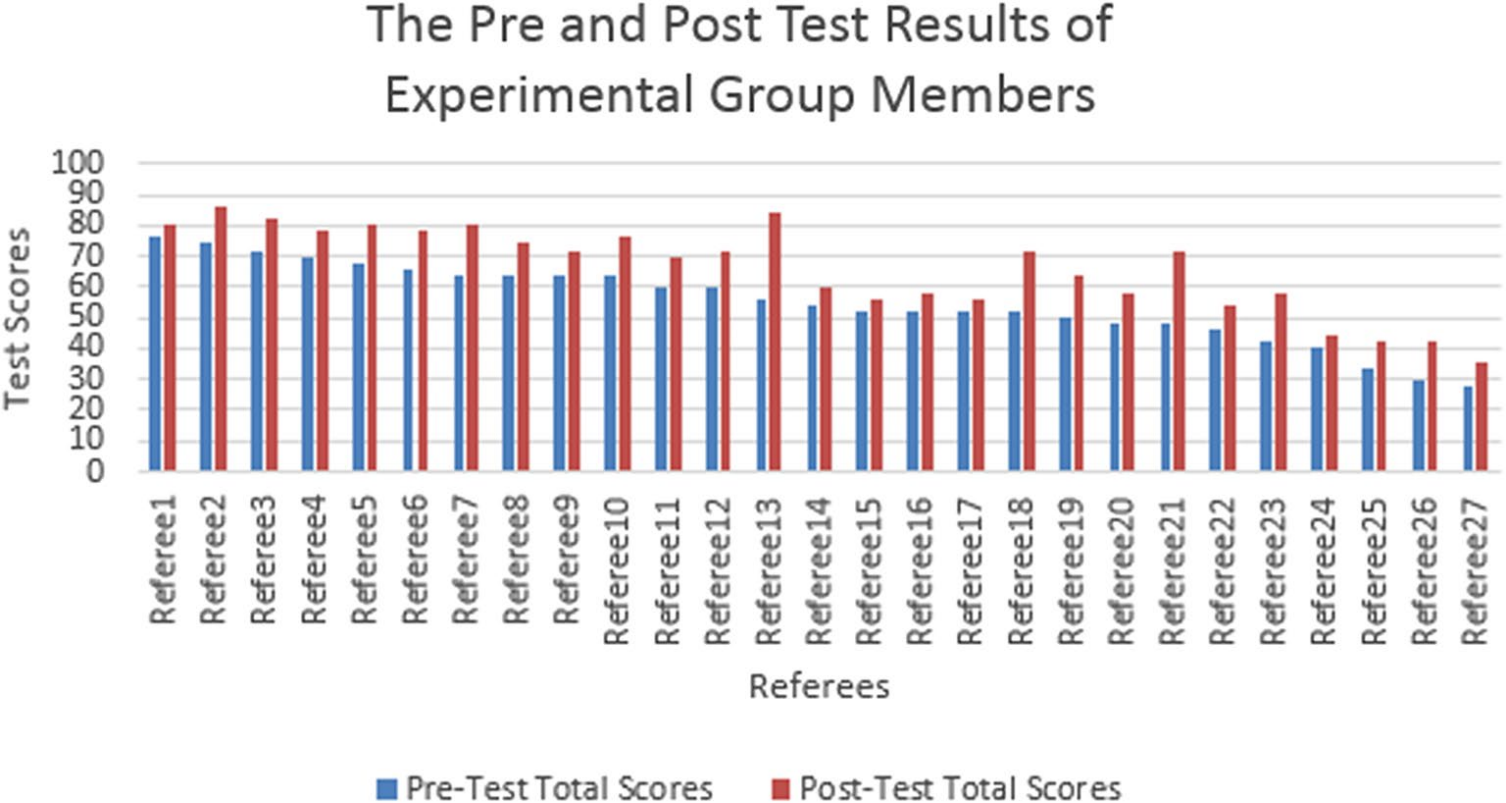
Total scores of the referees for experimental group in pre and post test

**Fig. 4:**
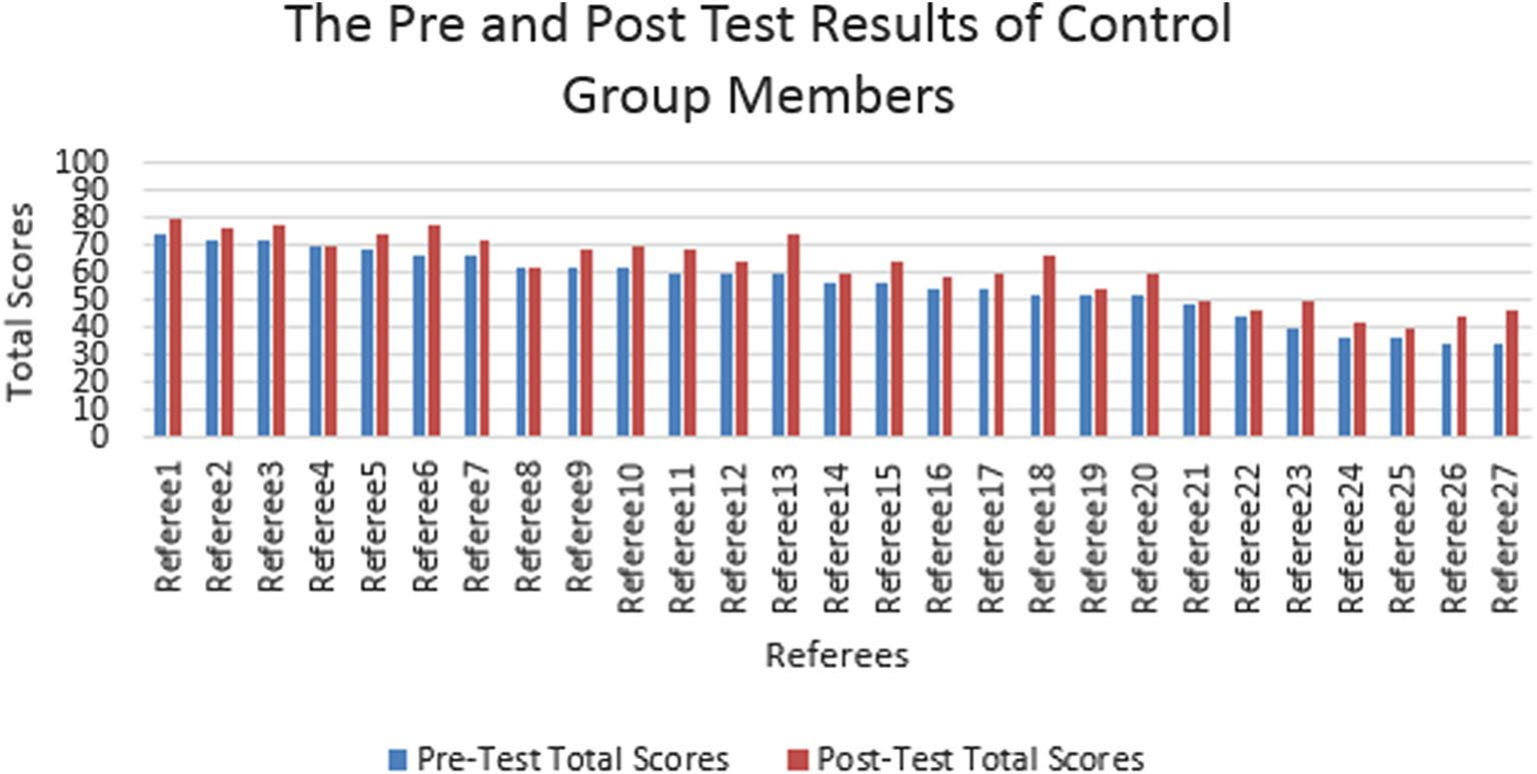
Total scores of the referees for control group in pre and post test

The pre-test was administered on 9 December 2014, at which there were 54 football referees as participants. The pre-test results of both groups are shown in Fig. 3 for the experimental group and Fig. 4 for the control group. The average scores of both groups in all parts are shown in Table 1.

**Table 1:** Average scores of both groups for all parts in pre-test

	Multiple choice (70)	Video (30)	Total (100)
Experimental group	38.3	16.8	55.1
Control group	38.4	17.3	55.7

Important points were considered during the data analysis. The first important point is that the average scores of both groups were very near to each other in every part of the exam. In other words, in both groups, the referees’ level of knowledge of the game was nearly identical at the beginning of the training program. The second important point was the number of successful referees in the exam. In real exams, 70 out of 100 is the threshold for referees to be successful. When the pre-test results were examined, from 27 individuals, 4 were found who were able to pass the exam in the experimental group (as shown in Fig. 3) and 4 individuals from 27 having passed the exam in the control group (as shown in Fig. 4). In total, from 54 referees, 8 were found to be successful.

At the end of the training period, a post-test was administered to a selected group of referees. This test was identical to the pre-test. The number and content of the questions were also identical because the difficulty level of the questions was to remain identical between the pre-test and the post-test in order to provide validity. The answers to the questions in the pre-test and the scores were not announced to the participants.

The post-test was administered on 6 January 2015. The post-test results of both groups are shown in Fig. 3 for the experimental group and Fig. 4 for the control group. The average scores for both groups in all parts are shown in Table 2.

**Table 2:** Average scores of both groups for all parts in post-test

	Multiple choice (70)	Video (30)	Total (100)
Experimental group	43.6	22.6	66.1
Control group	41.2	20.9	62.0

There were also important points when these quantitative data were analyzed, including the quantitative data obtained from the pre-test. First of all, the average scores of the experimental group in every part were higher than the average scores of the control group, meaning that the referees in the experimental group are more successful than the referees in the control group. The second important point is the number of successful referees in the exam. When the post-test results were examined, it was found that 15 individuals out of 27 passed the exam in the experimental group (as shown in Fig. 3), and 9 individuals out of 27 passed the exam in the control group (as shown in Fig. 4). In total, there were 24 successful referees out of the 54 referees.

## Discussion

There were several differences between the results of both the pre-test and the post-test.

Firstly, the number of successful referees in the post-test was higher than the number of successful referees in the pre-test. In the pre-test, there were 4 successful referees in the experimental group and 4 successful referees in the control group. Hence, there were 8 successful referees in total. In the post-test, there were 15 successful referees in the experimental group and 9 successful referees in the control group. Thus, there were 24 successful referees in total. These values showed that the digital game-based learning platform was more beneficial than traditional learning book because the number of referees who improve their knowledge level by using training platform outnumbered those referees using the LOG book.

The second difference was the average scores of both tests. For the experimental group, the average score for the multiple choice category was 38.3; for the video category, it was 16.8 and the average score in total was 55.1. For the control group, the average score for multiple choice category was 38.4, and for the video category it was 17.3. The average score in total was 55.7 in the pre-test. In the post-test, the average score for the multiple choice category was 43.6, and for the video category it was 22.6; the average score in total was 66.1 for the experimental group. The average score for the multiple choice category was 41.2, and for the video category it was 20.9; the average score in total was 62.0 for the control group. These numerical values showed that although the referees in the control group improved their scores, the referees in the experimental group increase their scores to greater extent than the control group in all parts of the exams.

When the members of the experimental group are compared to each other according to the number of completed game sessions, it can be easily seen that the players who have played more games have a higher level of development (as in Table 3).

**Table 3:** Number of completed games versus difference between pre-test and post-test

Participants	# of game sessions	Pre-test results	Post-test results	Difference
Participant 1	12	76	80	4
Participant 2	18	74	86	12
Participant 3	20	72	82	10
Participant 4	21	70	78	8
Participant 5	24	68	80	12
Participant 6	23	66	78	12
Participant 7	26	64	80	16
Participant 8	19	64	74	10
Participant 9	14	64	72	8
Participant 10	23	64	76	12
Participant 11	17	60	70	10
Participant 12	27	60	72	12
Participant 13	36	56	84	28
Participant 14	11	54	60	6
Participant 15	9	52	56	4
Participant 16	7	52	58	6
Participant 17	6	52	56	4
Participant 18	19	52	72	20
Participant 19	24	50	64	14
Participant 20	21	48	58	10
Participant 21	29	48	72	24
Participant 22	16	46	54	8
Participant 23	15	42	58	16
Participant 24	6	40	44	4
Participant 25	9	34	42	8
Participant 26	17	30	42	12
Participant 27	13	28	36	8

In addition to the above comparison, *two sample t-test* was applied to illustrate statistically to assess the training platform. When the value of the significance level was chosen to be 0.05 and assuming the variances to be equal to each other, the “*T*” value was calculated to be 3.45 and the “*p*” 0.001. According to the *t*-Table, the critical value was equal to 1.675 since population number is equal to 52 and the value of the significance level is equal to 0.05.

According to the results obtained from the *two sample t-test*, a significant difference can be seen between the population mean of the experimental group and the control group. This conclusion indicates that the knowledge of the members of the experimental group exceeded that of the members of the control group.

To strengthen the validity of the study, the Wilcoxon signed-rank test was used to understand whether a difference exists between the knowledge levels of members from both groups regarding the rules of football by analyzing both the pre-test and the post-test results.

For the pre-test results, there were 19 non-zero difference values (Table 4) from 27 pairs of scores. According to these 19 non-zero difference values, the positive summation value was equal to 100.5 and the negative summation value was equal to 89.5 which was taken as the absolute value of −89.5. Thus, our test statistic value equalled 89.5, which was the smaller value between the absolute value of the positive and negative summation values. According to the Wilcoxon signed-ranks table, the critical value is equal to 46 when the number of non-zero difference values is calculated as 19, and the value of the significance level is taken as 0.05. According to these numerical values, it failed to reject the null hypothesis, implying that there is no significant difference between the knowledge levels of members from either group with regard to the rules of the game at the beginning of the training period since the critical value was lower than the test statistic value.

**Table 4:** The difference values in pre-test and post-test

	Differences in pre-test	Differences in post-test
1	2	10
2	2	4
3	−2	8
4	2	6
5	2	8
6	2	12
7	−4	4
8	−2	6
9	−4	2
10	−2	8
11	−2	10
12	−2	−8
13	−4	−4
14	2	6
15	2	10
16	4	−2
17	−2	22
18	−4	8
19	−6	8
20		2
21		2
22		−2
23		−10
24		
25		
26		
27		

When the results of the post-tests were analyzed, 23 non-zero difference values (see Table 4) from 27 pairs of scores were found. According to these 23 non-zero difference values, the positive summation value equalled 204 and the negative summation value equalled 72. The latter result was taken as the absolute value of −72. Thus, our test statistic value was equal to 72. According to the Wilcoxon signed-ranks table, the critical value is equal to 73 when the number of non-zero difference values is calculated as 23 and the value of significance level is taken as 0.05. According to these results, it rejects the null hypothesis which means that there is a difference between the knowledge levels of both group members regarding the rules of the game at the end of the training period since the critical value is higher than the test statistic value. When the average scores of the referees in both groups were calculated, the average score of the experimental group was higher than the average score of the control group.

In addition to the above quantitative analysis, a set of interviews was conducted with five participants to learn about their opinions and experiences with the game in order to complete the qualitative part of the study.

As one interviewee stated:

Interview quotation: After playing the game, I observed that my ability to make true comments about the positions occurred during the football games was improved. Consequently, this feels wonderful, now I feel more engaged with the game.

As another interviewee commented:

Interview quotation: I think the game platform creates a structural competition between the participants to get a higher position in overall rankings. Also, I find the majority of the questions in the game platform was helpful to remind the rules of the football game.

As another interviewee stated:

Interview quotation: I think the most important feature of this system is easily accessible. Whenever I want, I can play the game. I do not need to wait for the conferences to see the positions in the matches. Furthermore, it is more enjoyable than the conferences, however, the contents of the questions should always be kept as updated. If I solve the same questions again and again, I may not want to play the game after a certain time.

One participant reported:

Interview quotation: There are nearly 200 referees in the conferences organized in every month. It is very hard to show our abilities among this crowded group, however, the game gives us a chance to show our abilities by listing the successful referees in overall ranking. If a referee has a good knowledge about the laws of the game, his name will be written on the list. Therefore, the managers can distinguish the successful referees.

Another participant declared that:

Interview quotation: We need to have an alternative resource to do practice on the laws of the football game, because, we have to study the rules by using only the LOG book and this book is not an interesting tool for education. This game brings us an innovation to study the rules.

To increase the validity of the qualitative part of the study, five retired first class football referees evaluated the answers of the interviews as positive, negative or neutral. The result of this evaluation is shown in Table 5.

**Table 5:** Evaluation of the retired referees

	Interview 1	Interview 2	Interview 3	Interview 4	Interview 5
Evaluator 1	Positive	Positive	Positive	Positive	Positive
Evaluator 2	Positive	Positive	Neutral	Positive	Positive
Evaluator 3	Positive	Positive	Neutral	Positive	Neutral
Evaluator 4	Positive	Positive	Positive	Positive	Positive
Evaluator 5	Positive	Positive	Neutral	Positive	Positive

According to the evaluation of the retired referees, the first, second, fourth and fifth interviews were found to be positive; however, three of the referees had evaluated the third interview as neutral. Hence, the participants are considered to have made positive comments about the system since most of the answers of the interviews were found as positive.

## Conclusions

Despite its exploratory nature, the main objective of this study is to explore the benefits of game-based learning while creating an assessment tool for the training of football referees in an enjoyable manner. To this end, an assessment-based training framework has been developed, which was designed with the idea to improve the decision-making abilities of football referees, particularly to interpret critical situations in the course of a match. In addition, the authors believe that it is important to propose a potential training program by investigating the knowledge levels of individuals when no official program is offered by the Turkish Football Federation.

The results which were obtained by statistical analysis suggest that the proposed platform is a beneficial tool to educate referees about the rules of the game. The current findings are important for the growing body of literature on the importance of educating referees. Taken together, these findings suggest a crucial role for game-based learning activities for the training of football referees which should highlight a new perspective to FIFA regarding the subject matter.

In addition to the quantitative parts of the study, initial interviews were conducted to develop the gaming platform. At the end of the study, validation interviews were conducted to capture the football referees’ experiences and opinions about the training framework.

Based on the validation interviews, we have indicated that this study has gone some way towards enhancing our understanding of the decision-making skills of referees based on the comments about the positions occurring during football matches. One interviewee suggests that these studies improve the self-confidence of the participants based on their progressions. This study has demonstrated, for the first time, that even a simple game element, such as the leader-board, is effective at engaging referees in the training program. Lastly, the majority of the participants found the platform beneficial as a complementary tool for studying, especially prior to the actual exams. The tool provides opportunities for novice football referees to follow new positions occurring throughout matches played all over the world. In fact of using the proposed system, more positions can be shown to football referees to improve their decision-making skills.

Although we had reached more than fifty novice and ten expert referees during this study, these findings may still be somewhat limited in terms of sample size. However, many attempts have been made to avoid this. We convinced many of the participants by explaining to them the importance of the study and by investing considerable effort in the collection of detailed data from football referees (i.e., over 60 h of direct interviewing time in addition to the associated data transcription).

To improve the quality of training, further research should also be conducted to provide a more immersive environment with computer-generated landscapes with a 3D virtual soccer field that could be based on virtual reality or augmented reality. Such a system would provide full simulation of a match in a virtual football field, which can provide the trainee a real life like a learning experience. A further study with more focus on referee training on such a medium that is based on the tracking of the positions and actions of a participant with continuous feedback on an artificial interactive computer-generated football field is therefore suggested.
